# The complete mitochondrial genome of *Flavoperla biocellata* Chu, 1929 (Plecoptera: Perlidae) and the phylogenetic analyses of Plecoptera

**DOI:** 10.7717/peerj.8762

**Published:** 2020-03-16

**Authors:** Yue Shen, Yu-Zhou Du

**Affiliations:** 1 School of Horticulture and Plant Protection & Institute of Applied Entomology, Yangzhou University, Yangzhou, Jiangsu, China; 2 Joint International Research Laboratory of Agriculture and Agri-Product Safety, the Ministry of Education, Yangzhou University, Yangzhou, China

**Keywords:** Flavoperla, Plecoptera, Mitochondrial genome, Phylogeny

## Abstract

Of the roughly 400 species of Perlidae in the world, most species are widely distributed in the northern hemisphere, but a few can be found in South Africa and South America. There are only five species in the genus *Flavoperla* of the family Perlidae in China. To gain a better understanding of the architecture and evolution of mitochondrial genome in *Flavoperla*, the entire mitochondrial genome (mitogenome) of a Chinese *Flavoperla biocellata* Chu, 1929 from family Perlidae (Insecta: Plecoptera) was sequenced. The 15,805-bp long mitochondrial genome of *F. biocellata* contained 37 genes, including 13 protein-coding genes (PCGs), 22 transfer RNA genes (tRNAs), two ribosomal RNA genes (rRNAs) and a putative control region (CR). The gene arrangement of *F. biocellata* was identical with that of other stoneflies and with the fly *Drosophila yakuba*. Most PCGs of *F. biocellata* used the standard ATN start codons and complete TAN termination codons. Twenty-one of the 22 tRNA genes exhibited cloverleaf secondary structures, but the dihydrouridine (DHU) arm of *trnSer* (AGN) was completely reduced. Phylogenetic analyses with both Bayesian inference (BI) and maximum likelihood methods (ML) generated similar topology, both supporting the monophyly of all stonefly families and the infraorder Systellognatha. The phylogenetic analysis based on mitochondrial genomic data from 30 stonefly species recovered a well-supported tree resolving higher-level relationships within Plecoptera. The northern hemisphere suborder Arctoperlaria divided into two groups, Euholognatha and Systellognatha. The southern hemisphere suborder Antarctoperlaria formed two clades: Eustheniidae+Diamphipnoidae and Austroperlidae+ Gripopterygidae; consistent with relationships proposed based on morphology. The final relationships within Plecoptera were recovered as (((Perlidae+(Perlodidae+Chloroperlidae))+(Pteronarcyidae+(Peltoperlidae+Styloperlidae))) +(Taeniopterygidae+(Capniidae+(Nemouridae+Notonemouridae))))+ (Gripopterygoidae+Eusthenioidae).

## Introduction

The Plecoptera (stoneflies) is an ancient order of aquatic insects that is an important bioindicator of water quality (William & Felmate, 1992). It is also important for the phylogenetic reconstruction of insects. Plecoptera contains two suborders, Antarctoperlaria and Arctoperlaria, including 16 extant families (Zwick, 1973). However, the phylogenetic relationship among the families of the Plecoptera is still unresolved (Gibson et al., 2004; Cameron, 2014). The area of origin and sister-group of Plecoptera are inconclusive (Zwick, 2009; Yoshizawa, 2011; Simon et al., 2012; Letsch & Simon, 2013; Song et al., 2016). In 2009, Zwick tried to compare the hypotheses concerning the placement of stoneflies based on the morphological evidence, but failed (Zwick, 2009). Most molecular studies support Dermaptera as the sister group of Plecoptera, however, the transcriptome data suggests that Plecoptera may be one of the basal branches of Polyneoptera, and sister to a clade composed of all other orders except Dermaptera and Zoraptera (Ishiwata et al., 2011; Yoshizawa, 2011; Letsch & Simon, 2013; Wu et al., 2014; Song et al., 2016). Low taxon sampling within Plecoptera which reduced the reliability of their proposed phylogenies (Ding et al., 2019). To develop a better understanding of the phylogeny of Plecoptera, we sequenced the mitochondrial genome of *F. biocellata*, which was the first species sequenced in the genus *Flavoperla* from the family Perlidae of the Arctoperlaria. There are only five species of *Flavoperla* in China, and thus the sequence of the mitochondrial genome of *F. biocellata* will help us understand the architecture and evolution of the genus and will supplement data on the Perlidae. Recently, the number of complete mitochondrial genome sequences available has increased dramatically due to the development of next-generation sequencing technology. But the information on the Perlidae is still limited, with only five complete mitochondrial genomes sequenced, two from *Kamimuria,* and one each from *Togoperla, Dinocras* and *Acroneuria*. Mitochondrial genomes have been used in phylogenetic and evolutionary studies of insects, including the Plecoptera (Chen & Du, 2017a; Chen & Du, 2017b; Chen & Du, 2018; Chen et al., 2018; Wu et al., 2014; Wang, Cao & Li, 2017; Wang et al., 2019). To date, over thirty mitochondrial genomes of Plecoptera have been sequenced and reported, and their nucleotide composition and gene arrangement are highly conserved and identical with that of *Drosophila yakuba* (Wei & Chen, 2011; Chen & Du, 2015; Chen, Wu & Du, 2016; Chen & Du, 2017a; Chen & Du, 2017b; Chen & Du, 2018; Chen et al., 2018; Huang et al., 2015; Zhou et al., 2016; Wu et al., 2014; Elbrecht et al., 2015; Wang, Ding & Yang, 2016a; Sproul et al., 2015; Wang, Cao & Li, 2017; Wang, Wang & Yang, 2016b; Wang et al., 2019). The mitochondrial genome of Plecoptera has been verified as a double strand circular molecule that is nearly 16 kb in length and contains 13 PCGs, 22 tRNAs, two rRNA and a large non-coding control region, similar to other mitochondrial genomes (Clary & Wolstenholme, 1985; Steinberg & Cedergren, 1994; Gibson et al., 2004; Jia & Higgs, 2007; Cameron, 2014; Chen & Du, 2018; Chen & Du, 2018; Wang et al., 2019). We analyzed the nucleotide compositions of mitochondrial organizations, codon usages, tRNA structures of *F. biocellata*, and performed phylogenetic analyses of Plecoptera on the strength of the nucleotide sequences of available stonefly mitochondrial genomes.

## Materials & Methods

### Sample collecting and DNA extraction

Fresh specimens of *F. biocellata* were collected from Liyang, Jiangsu Province China (31.43^∘^N, 119.48^∘^E) in 2018. No endangered or protected species were involved in the study. All the samples were collected by sweeping the trees near streams, and identified using a microscope to identify the samples and were then stored in 100% ethanol and held at −20 °C. Genomic DNA was extracted with the Column mtDNAout kit (Tianda, Beijing, China) from the identified adults and stored at −20 °C for subsequent PCR amplifications.

### PCR reaction and DNA sequencing

The mitochondrial genome of *F. biocellata* was amplified using a similar sequencing strategy to Chen & Du (2018). Firstly, the large and small ribosomal genes (*rrnL* and *rrnS*), *cox1* and *cox2* were amplified using four pairs of newly designed primers. Based on the four obtained sequences, two pairs of LA-PCR primers were designed to amplify two overlapping fragments covering the whole mitochondrial genome (*rrnL-cox2* and *cox1-rrnS*). The remaining gaps were filled with specially designed primers. LA-PCR amplifications were performed with LA Taq DNA polymerase (Takara, Japan) using the following conditions: 2 min of initial denaturation at 93 °C, followed by 40 cycles at 92 °C for 10 s; 30 s of annealing at 54 °C; and 8 min of elongation at 68 °C (20 cycles), which increased 20 s/cycle in the final 20 cycles; and 10 min of final elongation at 68 °C. Table 1 summarizes all primers used in this study. Electrophoresis with 1.0% agarose gels was used to separate the products according to gene size of PCR reactions. Axygen DNA Gel Extraction Kit was used to purify the PCR products. All obtained purified PCR fragments were sequenced using sanger sequencing method by Map Biotech Company (Shanghai, China).

**Table 1:** Primers used for the *Flavoperla biocellata* Chu, 1929 mitogenome.

**Primer name**	**Nucleotide sequence (5′–3′)**
FL-COI-16S-longF	GGTGTCTCCTCAATTCTAGGGGCAG
FL-ND5-3R	TATTTATAGCTGGATTAGGGGCT
FL012-1F	CGTAGTGGCTCATTTTCATTATG
FL012-1R	TAGCTTTAATTGTAAGTGTCTTGG
FL012-2F	AACTATCGGCCATCAATGATAC
FL012-2R	CTTCTTATTTGTTCTAGTCAGGCT
FL012-3F	TATTTTCCGTATTTGACCCATC
FL012-3R	AAATGTTGATCCGTATACAGCAT
LCO1490	GGTCAACAAATCATAAAGATATTGG
HCO2198	TAAACTTCAGGGTGACCAAAAAATCA
FL-16S-COI-longF	CTTGTCCAACCATTCATACCAGCC
FL-CO1-1R	CACTAATCAATTTCCAAACCCTC
FL033-1F	CAATACAATAACTCCTTCACTATAAAAT
FL033-1R	TAACTCATTTAGGGAGAAACCC
FL033-2F	AAATAATAGGGTATCTAATCCTAGTTT
FL033-2R	CTTGGTACACTGAAAATTAAGGCTG
FL033-3F	GTCCTTAATAGTTAAAGTTTATTGGC
FL033-3R	GATGGTACTCAAAAAGCATCTTC
16S-L(16SarL)	CGCCTGTTTATCAAAAACAT
16S-H(16SbrH)	CCGGTCTGAACTCAGATCACGT
FL-CB-1F	TGCTGCAGTCTTAGTTCACCTTC
FL-COI-16S-longR	CGCTGGGTGCATTATTTTATTGGG
FL014-1F	AAGGTAAAAATCTCTTTCACGC
FL-ND5-3F	ACCATCTCAACCCAATAAGATTC
FL-CB-1R	AAAGGATTGGCTGGAGTAAAATTAT
FL009-1F	AATAAAAACCACCATAAAGTTATTCT
FL009-1R	CATGAGCGGTGTAATGTATAGC
FL009-2F	TAAAACAGAAACAAATTTATTGAACT
FL-ND5-2F	TTGAGAATCCTTTATATTATGAATAATAGC
FL-ND5-1R	ATGATTTAGTGTATTTTATTGAATGGG

### Mitochondrial genome annotation

The mitochondrial genome was assembled with CodonCode Aligner (http://www.codoncode.com/aligner/). BioEdit was used to align all used mitochondrial genomes. Alignment with other published Plecoptera mitochondrial genomes were performed to identify the PCGs and rRNAs. ORF finder was used to examine the boundaries of the PCGs. The mitochondrial genome map was depicted by CGView (http://stothard.afns.ualberta.ca/cgview_server/). ARWEN (http://mbio-serv2.mbioekol.lu.se/ARWEN/) was used to detect the tRNAs and show their secondary structures. MEGA v. 6.0 was used to calculate the nucleotide composition. Composition skew values were obtained with AT-skew = [A–T]/[A+T] and GC-skew = [G–C]/[G+C]. The mitochondrial genome sequence of *F. biocellata* was added to GenBank under accession number MK905206.

### Phylogenetic reconstructions

In this study, 30 stonefly mitochondrial genomes were used for the phylogenetic analyses; the outgroup was the mayfly *Parafronurus youi* (Insecta Ephemeroptera) whose GenBank accession number is EU349015 (Table 2). MAFFT was used to align the amino acid sequences of the 13PCGs and concatenate them excluding the stop codons (Vaidya, Lohman & Meier, 2010; Katoh & Standley, 2013). PartitionFinder v.2.1.1 with BIC (Bayesian Information Criterion) was used to determine the best nucleotide substitution models (GTR+I+G) and the best partitioning schemes (partition by gene types) using a greedy search algorithm with unlinked branch lengths (Lanfear et al., 2016). BI analysis (Bayesian inferences) was conducted with MrBayes v.3.2.6, conditions are as follows: 20 million generations of runs sampled every 1,000 generations; four chains (three hot chains and one cold chain) with a burn-in of 25% trees (Ronquist & Huelsenbeck, 2003). Tracer v.1.5 was used to examine the stationarity of all runs (ESS > 200). Maximum likelihood (ML) analysis was performed using RAxML v.8 and had 1,000 bootstrap replicates (Stamatakis, 2014). FigTree v.1.4.2 was used to visualized all phylogenetic trees.

**Table 2:** Species of Plecoptera used in this study.

**Family**	**Species**	**Accessionnumber**	**Size(bp)**
Perlidae	*Kamimuria wangi*	KC894944	16,179
	*Kamimuria chungnanshana*	KT186102	15,943
	*Togoperla* sp.	KM409708	15,723
	*Dinocras cephalotes*	KF484757	15,666
	*Acroneuria hainana*	KM199685	15,804
	*Flavoperla biocellata* Chu, 1929	MK905206	15,805
Perlodidae	*Isoperla bilineata*	MF716959	15,048
	*Isoperla eximia*	MG910457	16,034
	*Pseudomegarcys japonica*	MG910458	16,067
	*Perlodes* sp.	MF197377	16,039
Chloroperlidae	*Sweltsa longistyla*	KM216826	16,151
	*Suwallia teleckojensis*	MF198253	16,146
Pteronarcyidae	*Pteronarcella badia*	KU182360	15,586
Peltoperlidae	*Cryptoperla sp.*	KC952026	15,633
	*Soliperla* sp.	MF716958	15,877
Styloperlidae	*Styloperla spinicercia*	KX845569	16,129
	*Styloperla* sp.	KR088971	15,416
Capniidae	*Mesocapnia arizonensis*	KP642637	14,921
	*Apteroperla tikumana*	KR604721	15,564
	*Capnia zijinshana*	KX094942	16,310
Nemouridae	*Nemoura nankinensis*	KY940360	16,602
Taeniopterygidae	*Doddsia occidentalis*	MG589787	16,020
	*Taeniopteryx ugola*	MG589786	15,353
Gripopterygidae	*Zelandoperla fenestrata*	KY522907	16,385
	*Antarctoperla michaelseni*	MK111413	16,069
Austroperlidae	*Klapopteryx armillata*	MK111414	14,870
Eustheniidae	*Neuroperla schedingi*	MK111415	16,882
Diamphipnoidae	*Diamphipnoa annulata*	MK111416	14,882
	*Diamphipnopsis sp.*	MK111417	16,714
Notonemouridae	*Neonemura barrosi*	MK111418	14,852

## Results

### Mitochondrial genome structure and nucleotide composition

**Figure 1:**
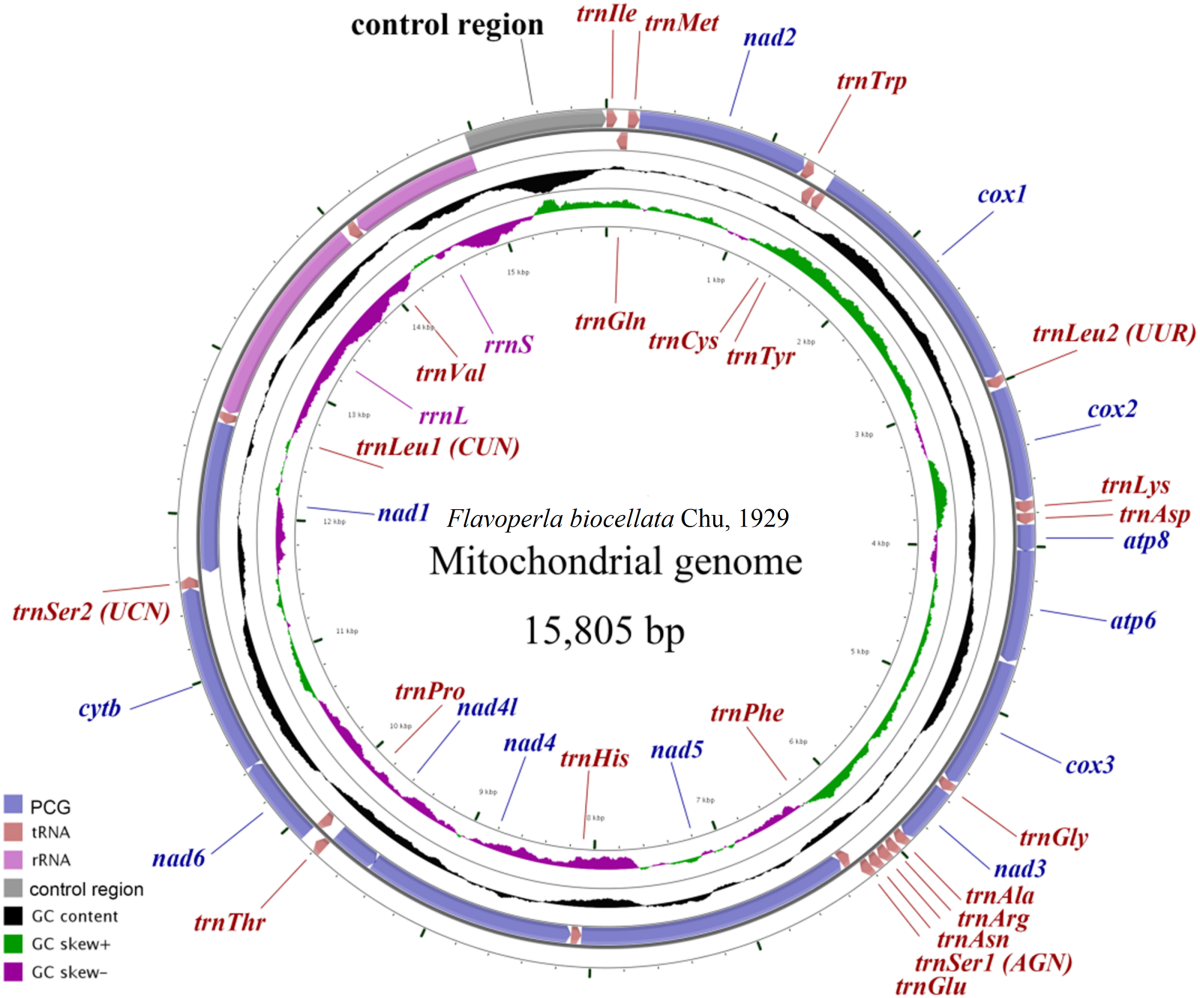
Mitochondrial maps of *Flavoperla biocellata* Chu, 1929. Genes outside the map are transcribed clockwise, whereas those inside the map are transcribed counterclockwise. The inside circles show the GC content and the GC skew. GC content and GC skew are plotted as the deviation from the average value of the entire sequence.

The *F. biocellata* mitochondrial genome was a double strand circular 15,805 bp molecule. The typical set of 37 genes was identified along with a 852 bp-long control region, of the total, 23 genes were found on the J-strand (majority strand) and 14 genes were on the N-strand (minority strand) (Fig. 1, Table 3). Altogether 54 overlapping nucleotides were found in 14 gene pairs, and their length ranged from 1 to 17 bp (Table 3). There were 84 intergenic nucleotides (IGNs) dispersed in 13 gene gaps; and the longest IGN was located between *16s (rrnL)* and *trnVal* and was 28 bp long, similar to other stoneflies. In *F. biocellata*, the whole mitochondrial genomes, the PCGs, tRNAs and rRNAs have very similar A+T contents:67.2%, 66.4%, 70.4%, and 70.6%, respectively (Table 3). A+T content of the 37 genes ranged from 53.0% in *trnTyr* to 83.6% in *trnGlu* (Table 3). The mitochondrial genome of *F. biocellata* has a positive AT-skew value and negative GC-skew value, biased for the A and C nucleotides.

**Table 3:** Mitochondrial genome structure of *Flavoperla biocellata* Chu, 1929.

**Gene**	**Position (bp)**	**Size (bp)**	**Direction**	**Intergenic Nucleotides (IGN)**	**Anti or Start/ StopCodons**	**A+T%**
*trnIle(I)*	1–65	65	Forward	0	GAT	67.7
*trnGln(Q)*	63–131	69	Reverse	−3	TTG	71.0
*trnMet(M)*	131–199	69	Forward	−1	CAT	66.7
*nad2*	200–1234	1,035	Forward	0	ATG/TAA	66.7
*trnTrp(W)*	1,233–1,300	68	Forward	−2	TCA	70.6
*trnCys(C)*	1,293–1,360	68	Reverse	−8	GCA	72.1
*trnTyr(Y)*	1,365–1,430	66	Reverse	4	GTA	53.0
*cox1*	1,414–2,963	1,550	Forward	−17	ATC/TT-	61.1
*trnLeu2(UUR)*	2,963–3,028	66	Forward	−1	TAA	68.2
*cox2*	3,039–3,726	688	Forward	10	ATG/T–	63.5
*trnLys(K)*	3,727–3,797	71	Forward	0	CTT	63.4
*trnAsp(D)*	3,800–3,868	69	Forward	2	GTC	81.2
*atp8*	3,869–4,030	162	Forward	0	ATT/TAA	72.8
*atp6*	4,024–4,701	678	Forward	−7	ATG/TAA	65.3
*cox3*	4,711–5,499	789	Forward	9	ATG/TAA	62.1
*trnGly(G)*	5,499–5,564	66	Forward	−1	TCC	80.3
*nad3*	5,565–5,918	354	Forward	0	ATT/TAG	65.5
*trnAla(A)*	5,917–5,984	68	Forward	−2	TGC	69.1
*trnArg(R)*	5,992–6,055	64	Forward	7	TCG	68.8
*trnAsn(N)*	6,055–6,124	70	Forward	−1	GTT	67.1
*trnSer1(AGN)*	6,125–6,191	67	Forward	0	GCT	61.2
*trnGlu(E)*	6,192–6,258	67	Forward	0	TTC	83.6
*trnPhe(F)*	6,258–6,325	68	Reverse	−1	GAA	70.6.
*nad5*	6,326–8,062	1,737	Reverse	0	GTG/TAA	68.3
*trnHis(H)*	8,063–8,127	65	Reverse	0	GTG	73.8
*nad4*	8,129–9,469	1,341	Reverse	1	ATG/TAA	68.5
*nad4l*	9,463–9,759	297	Reverse	−7	ATG/TAA	73.1
*trnThr(T)*	9,762–9,828	67	Forward	2	TGT	73.1
*trnPro(P)*	9,830–9,897	68	Reverse	1	TGG	73.5
*nad6*	9899–10,423	525	Forward	1	ATT/TAA	65.1
*Cytb*	10,423–11,556	1,134	Forward	−1	ATG/TAG	62.2
*trnSer2(UCN)*	11,555–11,624	70	Forward	−2	TGA	77.1
*nad1*	11,641–12,588	948	Reverse	16	TTG/TAG	68.5
*trnLeu1(CUN)*	12,590–12,656	67	Reverse	1	TAG	71.6
*rrnL*	12,659–14,009	1,351	Reverse	2		70.0
*trnVal(V)*	14,038–14,109	72	Reverse	28	TAC	65.3
*rrnS*	14,110–14,953	844	Reverse	0		71.1
CR	14,954–15,805	852		0		73.2

### PCG, tRNA and rRNA genes

**Figure 2:**
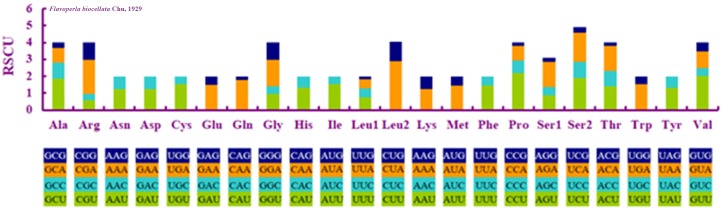
Relative synonymous codon usage (RSCU) in *Flavoperla biocellata* Chu, 1929. Codon families are indicated below the *X*-axis.

When compared with other stoneflies, each PCG of *F. biocellata* had a similar A+T content and length (Table 3). Eleven PCGs began with the standard start codon ATN (or ATT or ATG), however, for *nad5* GTG was the start codon, while for *nad1* it was TTG. Eleven PCGs of the mitochondrial genome had complete termination codons (TAA or TAG), but *cox1*, *cox2* terminated with an incomplete stop codon T. The RSCU (relative synonymous codon usage) value was calculated for *F. biocellata*, suggesting that the most frequently used codon was TTA (Leu), while CGC (Arg) was the least often used codon (Fig. 2, Table 4).

**Table 4:** Codons and RSCU of PCGs of *Flavoperla biocellata* Chu, 1929.

Codon	Count	RSCU	Codon	Count	RSCU	Codon	Count	RSCU	Codon	Count	RSCU
UUU(F)	238	1.47	UCU(S)	83	1.9	UAU(Y)	93	1.32	UGU(C)	35	1.52
UUC(F)	86	0.53	UCC(S)	41	0.94	UAC(Y)	48	0.68	UGC(C)	11	0.48
UUA(L)	301	2.88	UCA(S)	75	1.72	UAA(*)	0	0	UGA(W)	83	1.55
UUG(L)	121	1.16	UCG(S)	15	0.34	UAG(*)	0	0	UGG(W)	24	0.45
CUU(L)	79	0.76	CCU(P)	80	2.21	CAU(H)	53	1.31	CGU(R)	9	0.57
CUC(L)	55	0.53	CCC(P)	27	0.74	CAC(H)	28	0.69	CGC(R)	6	0.38
CUA(L)	55	0.53	CCA(P)	30	0.83	CAA(Q)	68	1.79	CGA(R)	32	2.03
CUG(L)	16	0.15	CCG(P)	8	0.22	CAG(Q)	8	0.21	CGG(R)	16	1.02
AUU(I)	216	1.52	ACU(T)	72	1.41	AAU(N)	99	1.25	AGU(S)	39	0.89
AUC(I)	69	0.48	ACC(T)	47	0.92	AAC(N)	59	0.75	AGC(S)	21	0.48
AUA(M)	138	1.46	ACA(T)	74	1.45	AAA(K)	54	1.23	AGA(S)	65	1.49
AUG(M)	51	0.54	ACG(T)	11	0.22	AAG(K)	34	0.77	AGG(S)	10	0.23
GUU(V)	127	2.03	GCU(A)	97	1.87	GAU(D)	43	1.25	GGU(G)	59	0.96
GUC(V)	27	0.43	GCC(A)	48	0.92	GAC(D)	26	0.75	GGC(G)	28	0.46
GUA(V)	63	1.01	GCA(A)	46	0.88	GAA(E)	59	1.49	GGA(G)	96	1.57
GUG(V)	33	0.53	GCG(A)	17	0.33	GAG(E)	20	0.51	GGG(G)	62	1.01

**Figure 3:**
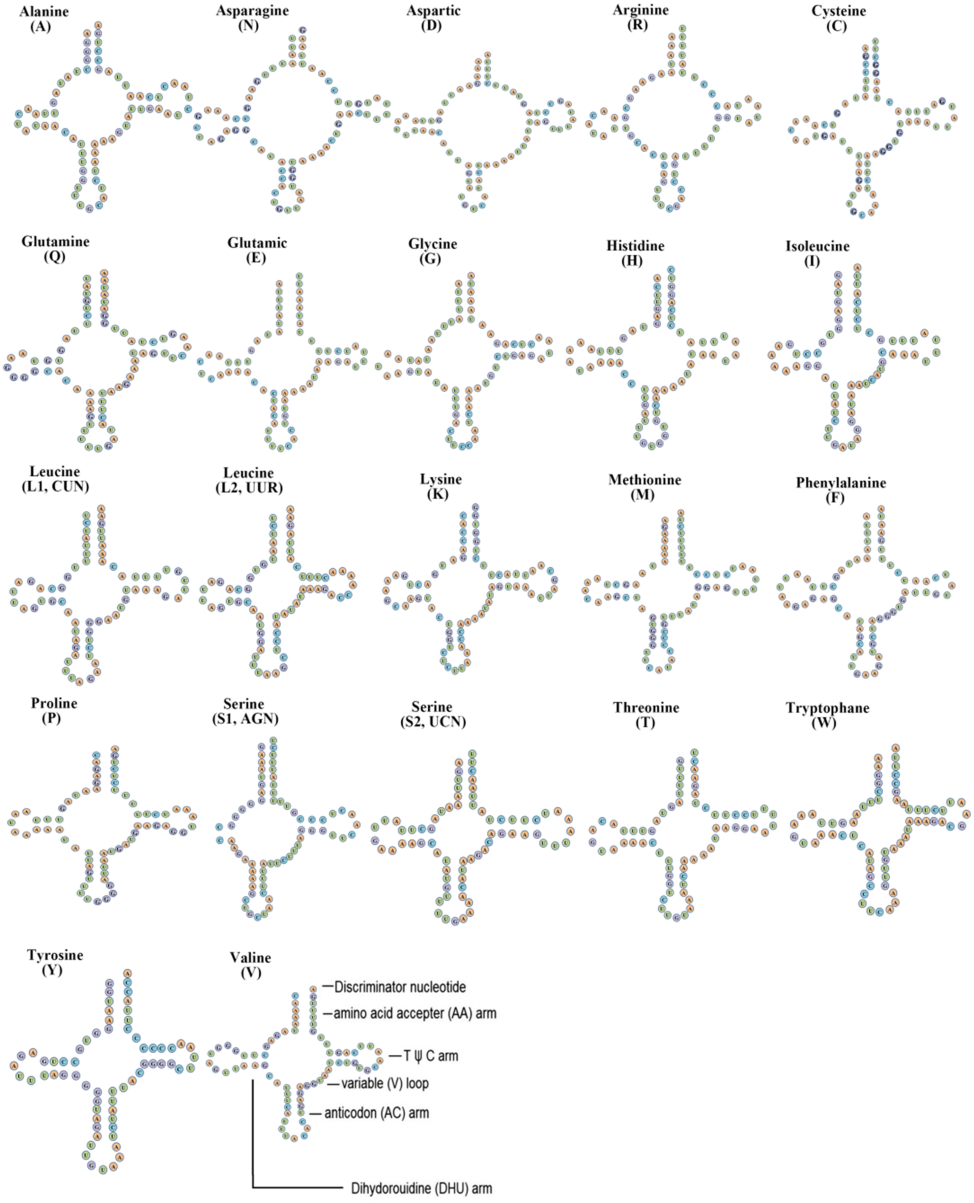
Inferred secondary structures of tRNAs from *Flavoperla biocellata* Chu, 1929. The tRNAs are labelled with their corresponding amino acids. Structural elements in tRNA arms and loops are illustrated as for trnV.

The *F. biocellata* mitochondrial genome had 22 tRNA genes, these tRNA genes were a total of 1,490 bp long with an average A+T content of 70.4% (similar to that in other stoneflies). Each tRNA gene ranged from 64 to 72 bp. Most tRNAs of *F. biocellata* had a typical cloverleaf secondary structure (Fig. 3), however, the dihydrouridine (DHU) arm of *trnSer* (AGN) was reduced to a small loop. Anticodons of all tRNAs in *F. biocellata* were identical with other species in Plecoptera. Thirty-three mismatched base pairs were detected in the tRNAs of *F. biocellata*, all of which were G-U pairs (Fig. 3). Two rRNAs in *F. biocellata* had a total length of 2,195 bp and an A+T content of 70.6%. The large ribosomal RNA gene (*rrnL*) was 1,351 bp with an A+T content of 70%, the small ribosomal gene (*rrnS*) was 844 bp long, with an A+T content of 71.1% (Table 3).

### The control region

In the mitochondrial genome of insects, the most variable region is the control region, which is difficult to sequence because of its complicated secondary structures. The control region of *F. biocellata* was 852-bp long with an A+T content of 73.2%. All stoneflies have the conserved control region and the location was between *rrnS* and *trnIle* (Fig. 1).

### Phylogenetic analyses

**Figure 4:**
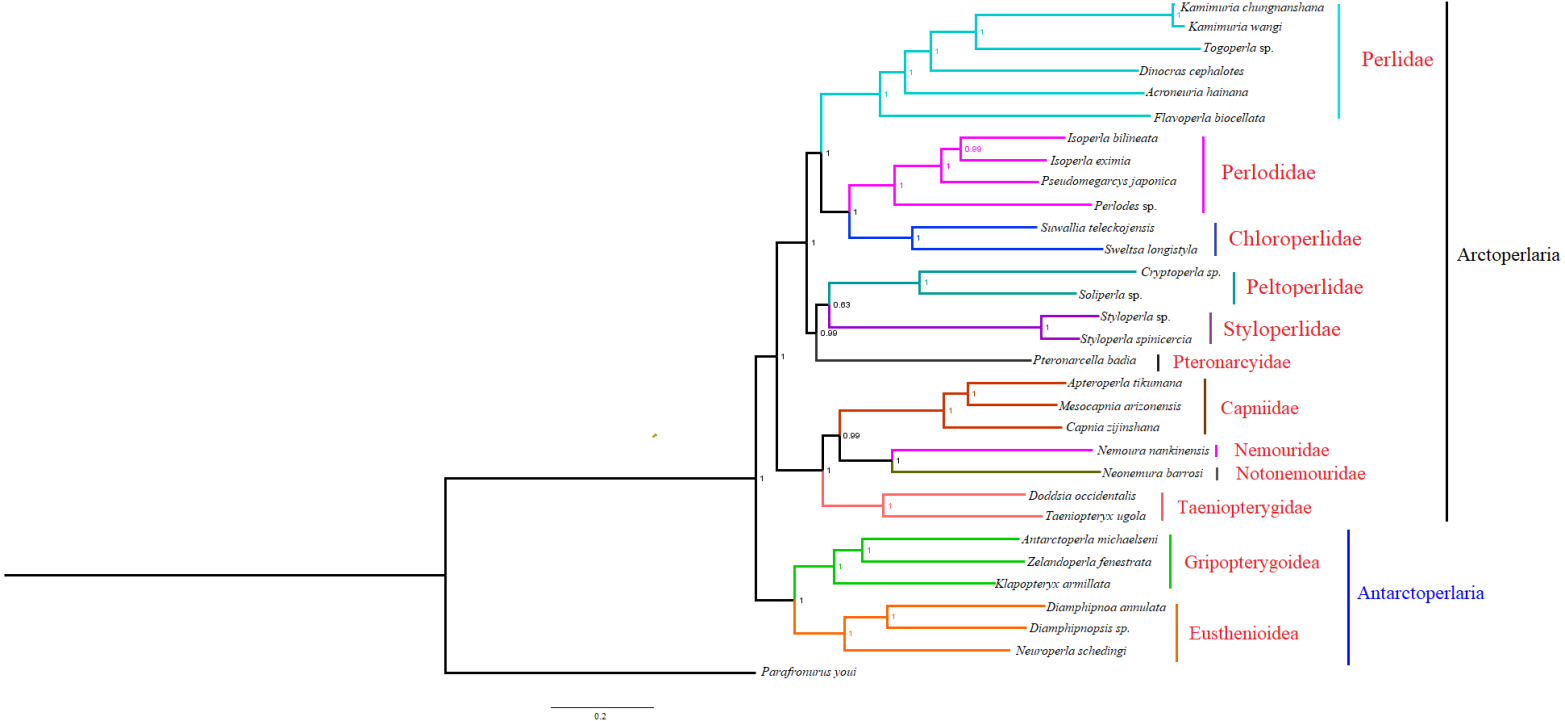
Phylogenetic relationships among stoneflies inferred by Bayesian inference. Numbers at the nodes are posterior probabilities. The family names are listed after the species.

**Figure 5:**
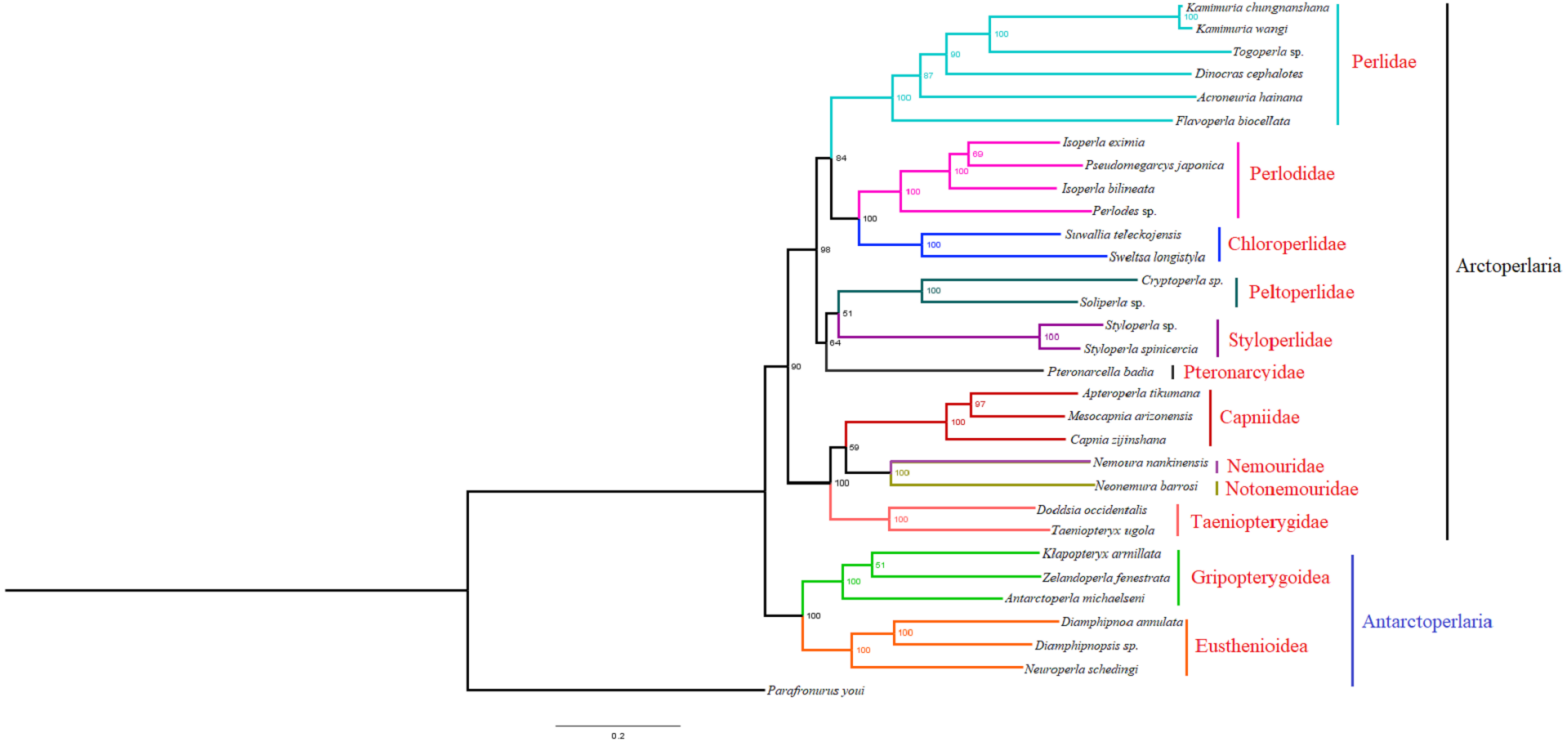
Phylogenetic relationships among stoneflies inferred by maximum likelihood analysis. Numbers at the nodes are bootstrap values. Family names are shown after the species.

Based on the concatenated sequences of 13 PCGs from 30 stonefly mitochondrial genomes, we reconstructed the phylogenetic relationship of Plecoptera; using the mayfly species *Parafronurus youi* as the outgroup. BI and ML were used to generate two phylogenetic trees which have generally identical topological structures (Figs. 4 and 5). In both analyses, the monophyly of each family is highly supported. The monophyly of suborders Antarctoperlaria and Arctoperlaria were consistently recovered in both trees. The phylogeny within Antarctoperlaria shows two superfamilies: Eusthenioidea (including Eustheniidae and Diamphipnoidae) and Gripopterygoidea (including Austroperlidae and Gripopterygidae), which is consistent with the study of Ding et al. (2019). In the suborder Arctoperlidae, the sister-group relationship between Nemouridae and Notonemouridae were consistently supported. In Systelognatha, the superfamily Perloidea is monophyletic; and the Perlodidae and Chloroperlidae are sister groups, which is congruent with recent studies (Chen & Du, 2018; Wang et al., 2019). The superfamily Pteronarcyoidea is monophyletic, with Styloperlidae being the sister group to the Peltoperlidae.

## Discussion

The mitochondrial arrangement of the *F. biocellata* mitochondrial genome was identical with other stoneflies and the model insect *Drosophila yakuba* (Clary & Wolstenholme, 1985), which was regarded as the ancestral mitochondrial genome due to the conserved gene order in non-insect hexapods (Nardi et al., 2003) and crustaceans (Crease, 1999). The nucleotide skew was consistent in Plecoptera and most other insects (Wei et al., 2010). The *trnSer* was had a small loop, which is a common phenomenon in stoneflies and other metazoans (Garey & Wolstenholme, 1989).

Zwick proposed two suborders: Antarctoperlaria and Arctoperlaria, and Arctoperlaria further divided into Systellognatha and Euholognatha (Zwick, 1973). In our study, the mitogenomic phylogeny strongly supported this classification (Figs. 4 and 5). In our study, the phylogeny within Antarctoperlaria supports the morphological phylogeny proposed by Zwick (Zwick, 2000). Ding et al. (2019) used four gene inclusion/exclusion datasets: protein-coding genes alone, with third codon positions (PCG123), PCGs without third positions (PCG12), PCGs including third positions plus rRNA genes (PCG123+RNA) and PCGs excluding third positions plus rRNAs (PCG12+RNA). Our study used the protein-coding genes alone, with third codon positions (PCG123) datasets and produced similar results. Our results also indicated the phylogeny within Antarctoperlaria showed two superfamilies: Eusthenioidea (including Eustheniidae and Diamphipnoidae) and Gripopterygoidea (including Austroperlidae and Gripopterygidae), which is consistent with Ding et al. (2019).

In both BI and ML, Nemouridae + Notonemouridae confirmed the northern origin of the currently southern hemisphere restricted Notonemouridae. Nemouridae currently is only found in the northern hemisphere, while Notonemouridae is only distributed in the southern hemisphere. Our phylogenetic analysis recovered the sister group relationship between Nemouridae and Notonemouridae, which supports the hypothesis that Notonemouridae was historically distributed in the northern hemisphere, but then migrated to the southern hemisphere and is now extinct from the northern hemisphere.

Uchida and Isobe proposed the relationships of (Pteronarcyidae + (Peltoperlidae + Styloperlidae)). Zwick then summarized the phylogeny of Systellognstha as ((Pteronarcyidae + (Peltoperlidae + Styloperlidae)) + (Perlidae + Chloroperlidae + Perlodidae)) (Zwick, 2000). Thomas et al. (2000) resolved the relationships as (Chloroperlidae + (Perlodidae + Perlidae)). Chen et al. reported (((Perlidae + (Chloroperlidae + Perlodidae)) + (Pteronarcyidae + Styloperlidae)) + Peltoperlidae) (Chen & Du, 2018). Our analyses showed (((Perlidae+(Perlodidae+Chloroperlidae)) + (Pteronarcyidae+(Peltoperlidae+Styloperlidae))). These different results may indicate that mitochondrial genomes are not a good data source for relationships within Systellognatha.

The difference in phylogenetic studies may have also resulted from the low numbers of samples; more sampling of the three families in future studies is expected to reconstruct a more robust phylogenetic relationship among the three families. Recent phylogenetic studies aimed to resolve the phylogeny of Plecoptera have demonstrated that the mitogenomic data were reliable and informative in inferring the inner phylogenetic relationships of Plecoptera. However, more comprehensive sampling especially for stoneflies from the southern hemisphere is needed to better resolve the mitochondrial phylogeny of Plecoptera.

## Conclusions

The entire mitochondrial genome of *F. biocellata* was sequenced and analyzed. The gene arrangement of *F. biocellata* was very conserved and identical with other stoneflies and the mitochondrial genome of *Drosophila yakuba*. The phylogenetic reconstructions with Bayesian inference (BI) and maximum likelihood methods (ML) had similar phylogenetic topology. More sequences should be obtained in future works to better resolve the phylogeny of Plecoptera.

## Additional Information and Declarations

### Data Availability

The following information was supplied regarding data availability: The mitogenome sequence of *Flavoperla sp.* is available at GenBank: MK905206.
